# Extracts from Dr. M. B. Campbell’s Annual Report

**Published:** 1897-06

**Authors:** 


DR. M. B. CAMPBELL, Medical Director of the Southern California State Asylum for the Insane and Inebriates, in his annual report states that there are over 100,000 persons in the United States afflicted with *narcomania*, and probably another 100,000 who have thus far been able to conceal this habit. He says that it is estimated that there are 10,000 opium, morphine and cocaine fiends in one of the leading cities of this coast.

